# MRI-based radiomics analysis and machine learning in the differentiation of endometriomas from hemorrhagic ovarian cysts

**DOI:** 10.1016/j.ejro.2026.100818

**Published:** 2026-09-12

**Authors:** Armin Doostparast, Zahra Alami, Sajjad Sadeghpour, Maryam Ghandhari, Mohamad Amin Bakhshali, Parvaneh Layegh

**Affiliations:** a Department of Radiology, School of Medicine, Mashhad University of Medical Sciences, Mashhad, Iran; b Eye Research Center, Mashhad University of Medical Sciences, Mashhad, Iran; c Nuclear Medicine Research Center, Mashhad University of Medical Sciences, Mashhad, Iran; d Student Research Committee, Faculty of Medicine, Mashhad University of Medical Sciences, Mashhad, Iran; e Department of Medical Informatics, School of Medicine, Mashhad University of Medical Sciences, Mashhad, Iran

**Keywords:** Endometrioma, Hemorrhagic ovarian cyst, Magnetic resonance imaging, Radiomics, Machine learning, Image processing

## Abstract

**Purpose:**

Endometriomas and hemorrhagic ovarian cysts (HOCs) are common benign ovarian lesions in reproductive-aged women. Their management differs substantially, and classic MRI signs, such as T1 hyperintensity and T2 shading, might overlap or introduce uncertainty. Therefore, we investigated the diagnostic performance of MRI-based radiomics combined with machine learning (ML) algorithms for differentiating ovarian endometriomas from HOCs.

**Methods:**

This prospective observational single-center study included patients with histopathologically confirmed endometriomas and HOCs. Lesions were manually segmented on T2-weighted MRI sequences, and radiomic features, including first-order, shape, texture, and gradient-based features, were extracted. Feature selection was performed using a nested cross-validation support vector machine recursive feature elimination (100-times per fold). Eight ML classifiers were trained and evaluated using nested ten-fold cross-validation for area under the curve (AUC), accuracy, sensitivity, specificity, and F1 score.

**Results:**

Out of 160 female patients (mean age: 31.3 ± 7.8 years), 89 (55.6%) were diagnosed with endometrioma. Five radiomic features were selected as the most discriminative predictors. Overall, the ensemble ML classifiers demonstrated better performance compared with the single-model classifiers (AUCs: 0.97–0.99 vs. 0.87–0.96). Extra Trees (ET) demonstrated the highest predictive performance (AUC = 0.99 [0.97–1.00], accuracy = 96%, sensitivity = 96%, specificity = 95%), which was significantly better than all of the single-model classifiers (DeLong’s test *P* < 0.04).

**Conclusions:**

MRI-based radiomics combined with ML algorithms demonstrates excellent performance in differentiating endometriomas from HOCs. This technique may hold clinical significance and, pending external validation, boost diagnostic confidence, decrease unnecessary surgeries, and aid in better patient management.

## Highlights

• Differentiating endometriomas from hemorrhagic ovarian cysts on MRI remains challenging due to overlapping signal characteristics.
• An Extra Trees classifier using five T2-weighted radiomic features achieved an area under the curve of 99%, a sensitivity of 96%, and a specificity of 95%.
• MRI-based radiomics with machine learning provides a non-invasive, objective approach for distinguishing endometriomas from hemorrhagic ovarian cysts.

## 1 Introduction

Endometriomas and hemorrhagic ovarian cysts (HOCs) appear as two of the most common benign conditions in women of reproductive age. Despite their benign nature, distinguishing these lesions accurately is essential because their treatment approaches and long-term effects vary substantially [1,2]. Endometriomas are ovarian cysts linked to endometriosis, a chronic inflammatory condition marked by ectopic endometrial tissue that can cause ongoing pelvic pain, infertility, and disease progression if untreated [1]. Conversely, HOCs are usually functional cysts associated with ovulation, often resolving naturally over time and typically managed conservatively rather than surgically [3]. Therefore, precise preoperative identification of these lesions is not only vital for guiding the correct treatment and preventing unnecessary surgeries, but also, in the case of endometrioma, for identifying other lesion sites that may indicate disease progression and associated complications [4,5].

Magnetic resonance imaging (MRI) is considered one of the most precise imaging methods for assessing complex adnexal lesions. It offers excellent soft-tissue contrast and allows detailed analysis of cyst contents using various pulse sequences [6,7]. Typical MRI signs of endometriomas include hyperintensity on T1-weighted images and low signal on T2-weighted images, known as the T2 shading sign, which indicates the presence of concentrated blood breakdown products within the cyst. These signal changes result from repeated cyclic hemorrhage in ectopic endometrial tissue, leading to high protein and iron levels that reduce T1 and T2 relaxation times [2,6,8]. T2-weighted imaging in MRI is especially crucial for identifying ovarian cystic lesions because changes in signal intensity often indicate chronic hemorrhage, clot formation, or protein buildup [9,10]. These characteristics offer important insights into the internal structure and persistence of the cysts and can be a significant source of quantitative radiomic data [2]. Although conventional MRI features are well known, there is still some diagnostic overlap between endometriomas and HOCs, especially when typical signs are missing or unusual. HOCs can sometimes look similar to endometriomas on imaging, particularly when blood products are at various stages of breakdown. Consequently, differentiating these two conditions based only on standard MRI signals can be difficult in everyday clinical practice [2,8].

Radiomics involves extracting a large number of quantitative features from images, capturing subtle patterns related to lesion heterogeneity, texture, and spatial intensity that may not be visible to the naked eye [11]. When used with machine-learning (ML) algorithms, these features can create predictive models capable of differentiating various pathological conditions. In ovarian imaging, early studies suggest that MRI-based texture analysis and radiomic feature extraction may better distinguish endometriomas from HOCs than traditional qualitative signal assessment, underscoring their potential as a non-invasive diagnostic tool [10,12]. Therefore, the present study aimed to investigate the diagnostic performance of radiomic features extracted from T2-weighted MRI images and to evaluate the ability of ML models to distinguish between these two clinically important ovarian lesions accurately.

## 2 Materials and Methods

### 2.1 Study design

This prospective observational study was conducted at a tertiary referral hospital between 2024 and 2026. The local institutional review board approved the study protocol (approval code: IR.MUMS.MEDICAL.REC.1401.566). The study procedures and goals were explained to all participants, and subsequently, written informed consent was obtained from all of them. All study procedures were conducted in accordance with the tenets of the Declaration of Helsinki.

### 2.2 Study population

**Fig. 1:**
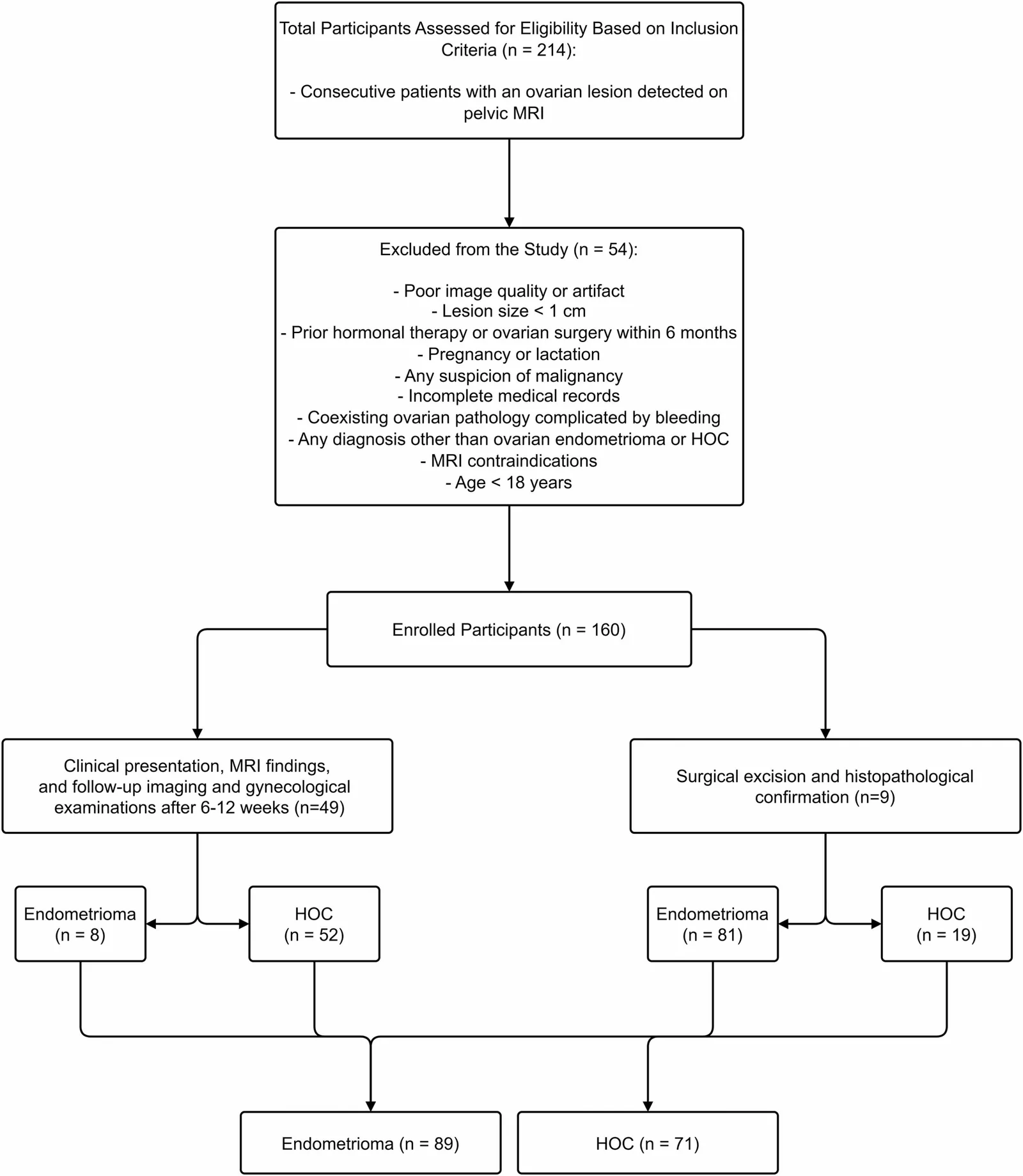
Flow diagram of patient enrollment and reference standards used to establish the diagnosis of ovarian endometrioma and hemorrhagic ovarian cyst (HOC).

This study included two groups of consecutive patients with a confirmed diagnosis of ovarian endometrioma and HOC based on histopathological examination and follow-up imaging and gynecological findings after 6–12 weeks. Exclusion criteria were as follows: (1) poor image quality or artifacts; (2) lesion size < 1 cm; (3) prior hormonal therapy or ovarian surgery within 6 months; (4) pregnancy or lactation; (5) suspicious features for malignancy; (6) incomplete histopathological or follow-up data; (7) coexisting ovarian pathology complicated by bleeding; (8) any diagnosis other than ovarian endometrioma or HOC; (9) MRI contraindications; and (10) age < 18 years. Fig. 1 illustrates a flow diagram of patient enrollment and the reference standards used to establish the diagnosis of ovarian endometrioma and HOC.

### 2.3 MRI protocol

All pelvic MRI examinations were performed using a 1.5-Tesla system (Siemens Symphony, Siemens Healthineers, Erlangen, Germany). Imaging was conducted with the patient in the supine position using a phased-array pelvic coil. The MRI protocol included non-contrast-enhanced sequences, specifically axial T1-weighted fat-suppressed (T1-FS) (TR = 500 ms, TE = 15 ms, slice thickness = 4 mm, FOV = 230 mm, and matrix = 256 × 256) and T2-weighted (T2-W) (TR = 4000 ms, TE = 90 ms, slice thickness = 4 mm, FOV = 230 mm, and matrix = 256 × 256) images covering the pelvis from the iliac crests to the pelvic floor.

The T1-FS sequence was obtained to evaluate hemorrhagic components and detect T1 hyperintense lesions characteristic of blood products. The T2-W sequence was used to assess lesion morphology, internal architecture, and the presence of the T2 shading and T2 dark spots signs commonly associated with endometriomas. All images were reviewed by an experienced radiologist during the follow-up examinations to confirm lesion characteristics, monitor the resolution of pathology, and ensure diagnostic consistency. Consistent with a previous study, the acquired T2-W images of preoperative MRI were subsequently used for lesion segmentation and radiomics feature extraction [13], as T2-W imaging was considered most suitable for characterizing the internal architecture and signal characteristics of these lesions.

### 2.4 Image processing and segmentation

All MRI data were retrieved from the institutional Picture Archiving and Communication System (PACS) and exported in Digital Imaging and Communications in Medicine (DICOM) format. Prior to analysis, all images were anonymized to remove patient identifiers. Lesion segmentation was performed manually by an experienced radiologist who was blinded to the final diagnosis. For each patient, a two-dimensional region of interest (ROI) was delineated along the margin of the lesion on the axial slice demonstrating the largest cross-sectional area of the cyst on T2-W images. Care was taken to include the entire cystic component of the lesion while avoiding adjacent ovarian tissue and surrounding pelvic structures. Before radiomics feature extraction, image preprocessing was performed to improve data consistency. Because features were extracted from a 2D ROI within the original 3D volumetric data, in-plane resampling was applied to standardize pixel spacing to 1 × 1 mm using B-spline interpolation. Intensity normalization was then conducted using z-score standardization, calculated from the mean and standard deviation of voxel intensities within the segmented 2D ROI. The segmented and preprocessed ROIs were subsequently used for radiomics feature extraction and ML analysis.

### 2.5 Radiomics feature extraction and feature selection

A total of 107 radiomic features were extracted from the segmented lesions on T2-W images using MATLAB R2020b and Python 3.11 (Pyradiomics library version 3.0.1). These features encompassed seven distinct categories: first-order statistics, shape features, Gray Level Co-occurrence Matrix (GLCM) features, Gray Level Size Zone Matrix (GLSZM) features, Gray Level Run Length Matrix (GLRLM) features, Neighboring Gray Tone Difference Matrix (NGTDM) features, Gray Level Dependence Matrix (GLDM) features, and Histogram of Oriented Gradients (HOG) features. HOG features were computed in MATLAB R2020b, while all other radiomics features were computed in Pyradiomics. These HOG features were included to capture edge orientation and local structural patterns not represented by first-order or gray-level co-occurrence-based features. A complete list of all extracted features with their corresponding categories is provided in Supplementary Table S1.

Feature selection was performed using support vector machine recursive feature elimination (SVM-RFE) with 100 bootstrap iterations, performed independently within each training fold of the outer ten-fold cross-validation (nested cross-validation design), to prevent information from the held-out test folds from influencing feature selection and to identify the most robust and informative features for distinguishing endometriomas from HOCs. The selection frequency of each feature was recorded within each fold and averaged across the 10 outer folds, and features with the highest mean selection frequencies were considered the most stable and discriminative. A pre-specified target of five features was set prior to analysis, based on the sample size and the goal of maintaining a conservative feature-to-sample ratio. As shown in Table 1, five features demonstrated the highest selection frequencies: coefficient of variation (CV) (frequency: 0.990 ± 0.011), GLCM homogeneity (0.848 ± 0.032), GLCM correlation (0.782 ± 0.036), standard deviation of the HOG feature vector (0.622 ± 0.049), and GLCM energy (0.618 ± 0.065). These five selected features were subsequently used as input for machine learning (ML) classification.

**Table 1:** Summary statistics of feature selection results using nested SVM-RFE across 10 outer cross-validation folds (100 bootstrap iterations per fold).

**Order**	**Feature**	**Type**	**Explanation**	**Frequency**
1	CV	First-order feature (histogram analysis)	Measures relative dispersion of voxel intensities by normalizing the standard deviation to the mean intensity, reflecting intra-lesional heterogeneity independent of absolute signal level	0.990 ± 0.011
2	GLCM Homogeneity	Second-order texture feature	Quantifies the uniformity of gray-level distributions by emphasizing local similarity between neighboring voxels; higher values indicate more homogeneous textures	0.848 ± 0.032
3	GLCM Correlation	Second-order texture feature	Assesses the linear dependency of gray-level values between neighboring voxels, reflecting the spatial correlation structure within the region of interest	0.782 ± 0.036
4	SD of HOG feature vector	Gradient-based texture feature	Represents the variability of local gradient orientations, capturing edge complexity and structural irregularity within the image	0.622 ± 0.049
5	GLCM Energy	Second-order texture feature	Quantifies the uniformity of squared gray-level co-occurrence values, reflecting the textural regularity and orderliness of voxel intensity pairs within the region of interest	0.618 ± 0.065

SVM-RFE: support vector machine recursive feature elimination, CV: coefficient of variation, GLCM: gray-level co-occurrence matrix, SD: standard deviation, HOG: histogram of oriented gradients. Selection frequencies reflect the mean ± SD of within-fold SVM-RFE selection frequency (100 bootstrap iterations per fold), averaged across the 10 outer cross-validation folds, to avoid information leakage between feature selection and model evaluation (nested cross-validation design).

### 2.6 Statistical analysis and ML

All statistical analyses and ML modeling were performed and visualized using Python (version 3.11). After feature selection, the five most discriminative radiomic features were subsequently used as input for ML classification. A stratified nested ten-fold cross-validation was applied during model training based on the distribution of the target variable to avoid the impact of inter-fold variability on final outcomes. Feature scaling was performed using standardization (Z-score normalization) fit on each training fold and applied to the corresponding test fold prior to model training. To prevent information leakage from the test folds, hyperparameter tuning was performed for all classifiers within each training fold via a nested inner cross-validation loop, using appropriate search strategies (grid/random search per algorithm) [14].

**Fig. 2:**
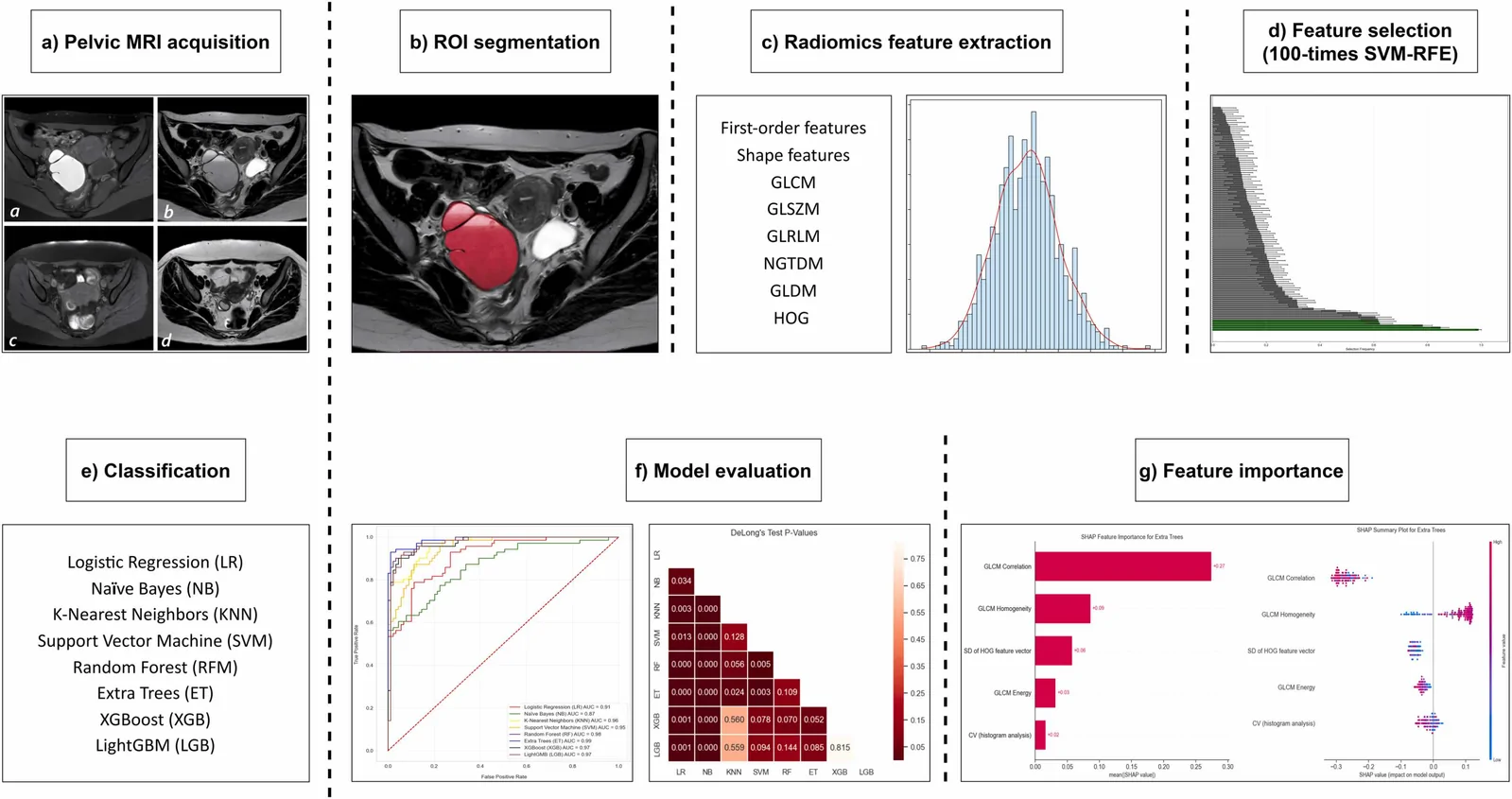
Flow Chart Diagram (a) Image acquisition/ b) ROI segmentation/ c) Radiomics feature extraction/ d) Feature selection/ e) Classification/ f) Model evaluation/ g) Feature importance.

Eight machine learning classifiers were developed and compared for their ability to distinguish endometriomas from hemorrhagic ovarian cysts: Logistic Regression (LR), Naïve Bayes (NB), K-Nearest Neighbors (KNN), Support Vector Machine (SVM), Random Forest (RF), Extra Trees (ET), XGBoost (XGB), and LightGBM (LGB). Model performance was evaluated using multiple metrics, including area under the receiver operating characteristic curve (ROC-AUC), area under the precision-recall curve (PR-AUC), accuracy, F1 score, precision (positive predictive value or PPV), sensitivity (recall), specificity, and Brier score. Confidence intervals (95% CI) for ROC-AUC and PR-AUC were calculated using bootstrap resampling with 1000 iterations utilizing the MLStatKit library (version 0.1.7). No a priori sample size or power calculation was performed because patients were consecutively enrolled during the predefined study period. The precision of the performance estimates was assessed using 95% CI. All performance metrics were reported as weighted averages to account for any class imbalance, and given the mild degree of class imbalance, no resampling technique was applied. Probabilistic performance and model calibration were evaluated using the Brier score, which quantifies the accuracy of predicted probabilities rather than binary classifications. Subsequently, pairwise comparisons of ROC-AUCs between all eight ML classifiers were performed using DeLong's test. The resulting P-values were then corrected for multiple comparisons using the Benjamini-Hochberg false discovery rate (FDR-BH) procedure. Lastly, the relative contribution of each feature to model performance was assessed using SHapley Additive exPlanations (SHAP) analysis. A P-value less than 0.05 was considered statistically significant across all analyses. A comprehensive review of the study workflow is depicted in Fig. 2.

## 3 Results

**Fig. 3:**
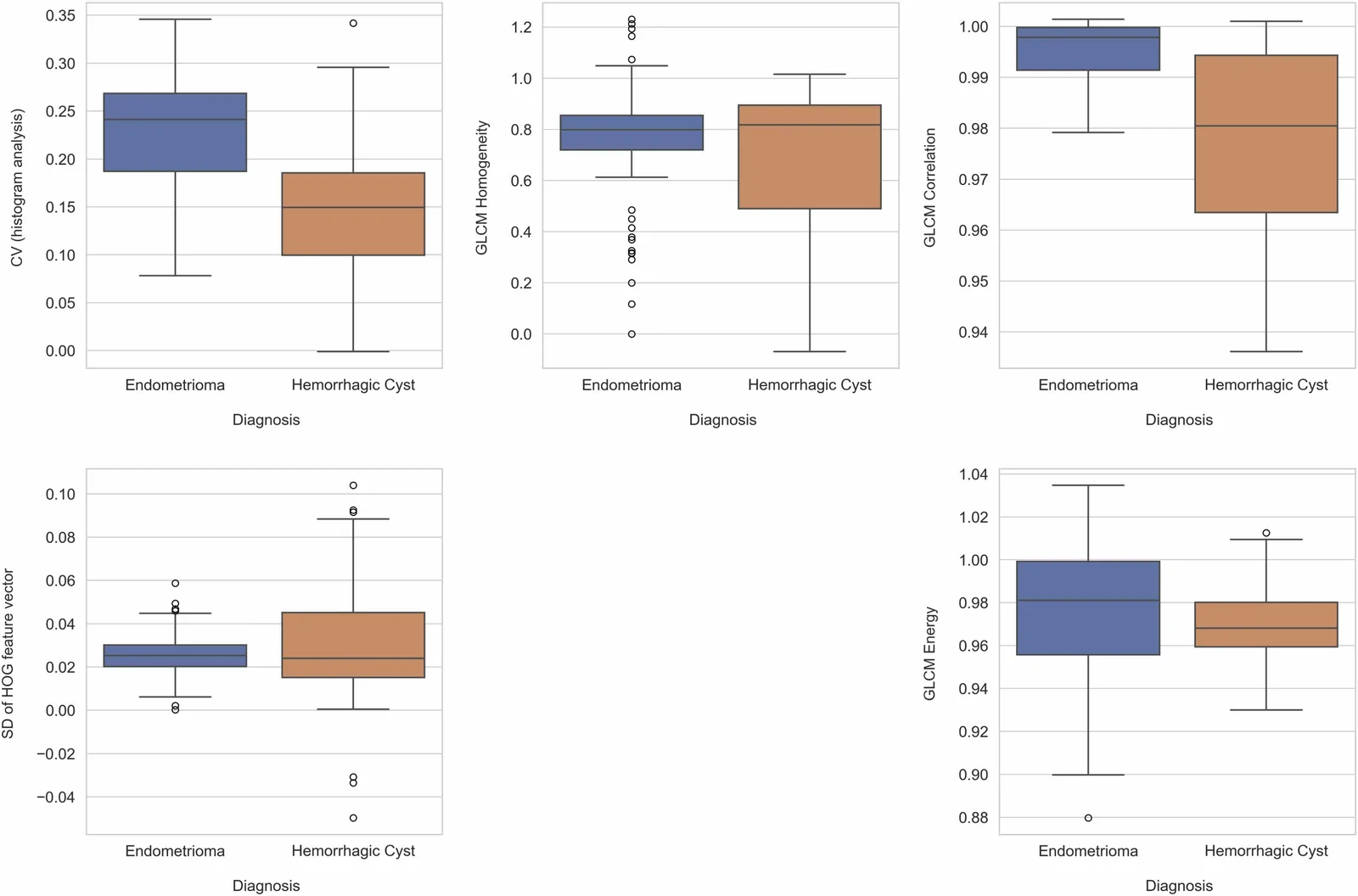
Box-and-whisker plots representing the distribution of selected radiomics features between the endometrioma and hemorrhagic ovarian cyst (HOC) groups. (CV: coefficient of variation, GLCM: gray-level co-occurrence matrix, SD: standard deviation, HOG: histogram of oriented gradients).

**Fig. 4:**
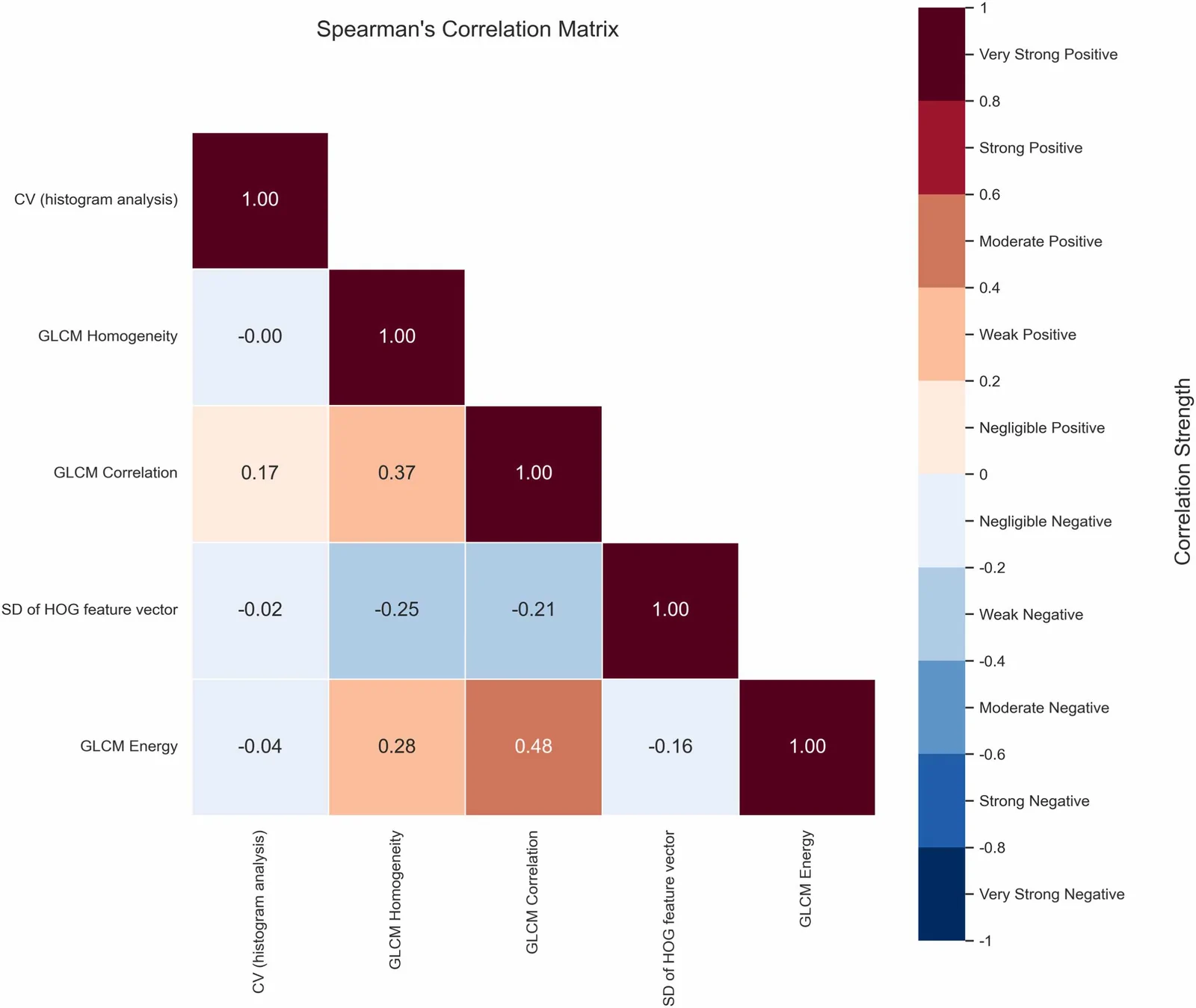
The correlation matrix illustrates the correlation of MRI-based radiomics. The values within the cells stand for Spearman’s rank correlation coefficient (ρ) (CV: coefficient of variation, GLCM: gray-level co-occurrence matrix, SD: standard deviation, HOG: histogram of oriented gradients).

This study included a total of 160 female patients, 89 (55.6%) diagnosed with ovarian endometrioma and 71 (44.4%) with HOC. The mean age of patients was 31.3 ± 7.8 years, with no statistically significant difference between the two groups (*P* = 0.15). Fig. 3, Fig. 4 represent the distribution of selected radiomics between the endometrioma and HOC groups and the correlation matrix of these MRI-based radiomics, respectively. Pairwise Spearman's correlation coefficients among the five selected features ranged from −0.25 to 0.48, indicating weak-to-moderate inter-feature correlation, with the strongest association observed between GLCM correlation and GLCM energy (ρ = 0.48) (Fig. 4).

**Fig. 5:**
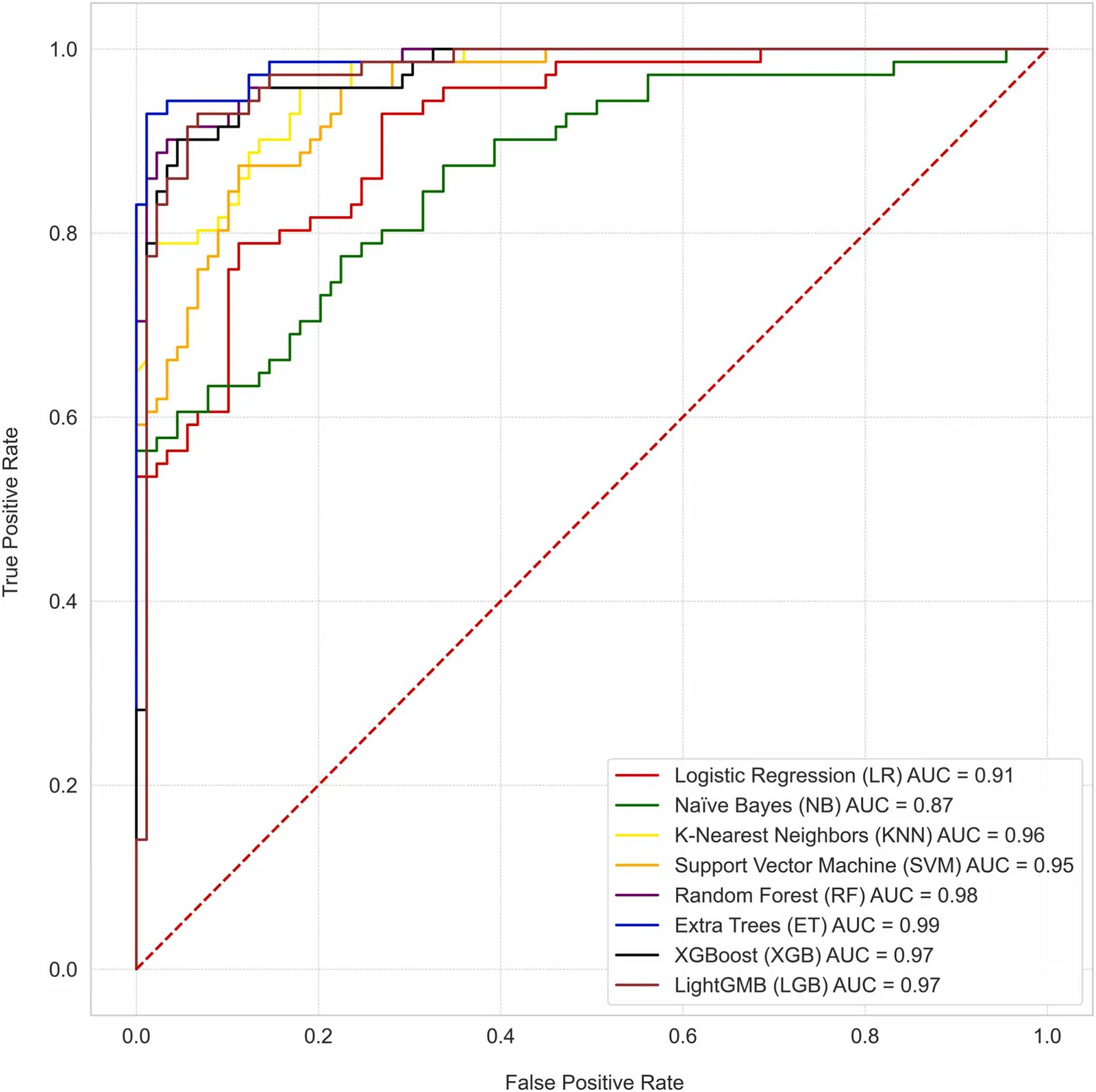
Receiver operating characteristic (ROC) curves of the machine learning (ML) classifiers used to distinguish endometrioma and hemorrhagic ovarian cyst (HOC) (AUC: area under the curve).

**Fig. 6:**
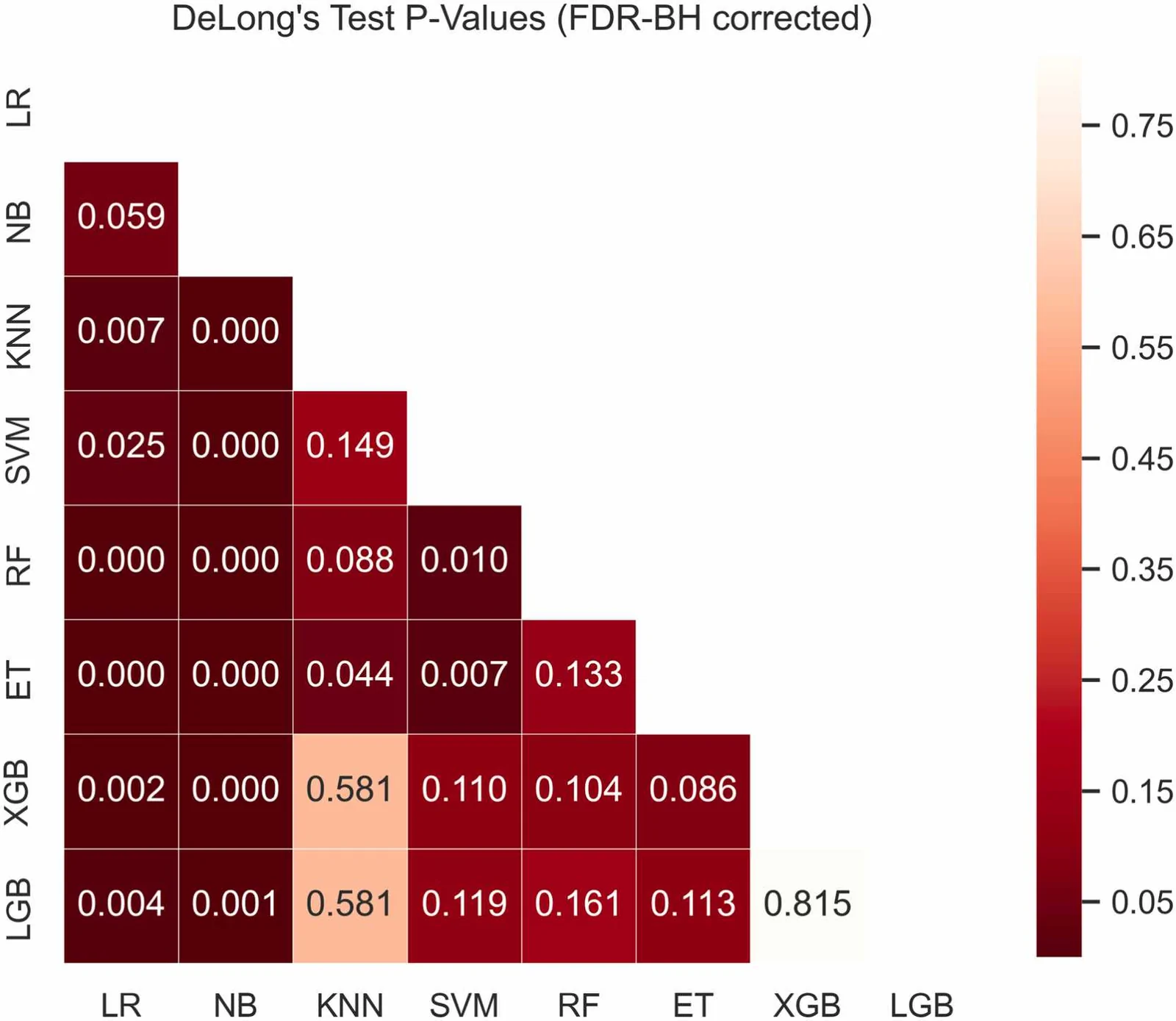
Heatmap of pairwise DeLong's test P-values, corrected for multiple comparisons using the Benjamini-Hochberg false discovery rate (FDR-BH) procedure, comparing the ROC-AUC of the eight machine learning classifiers (LR, logistic regression; NB, Naïve Bayes; KNN, K-nearest neighbors; SVM, support vector machine; RF, random forest; ET, extra trees; XGB, XGBoost; LGB, LightGBM).

Table 2 demonstrates the predictive ability of eight ML algorithms to distinguish endometrioma and HOC, with their corresponding ROC curves depicted in Fig. 5. It was found that the overall diagnostic performance of these algorithms was very high. Overall, the ensemble (ET, RF, XGB, LGB) ML classifiers demonstrated a higher ROC-AUC compared with the single-model (KNN, SVM, LR, NB) classifiers (0.97 – 0.99 vs. 0.87 – 0.96). The NB classifier exhibited the lowest predictive performance (AUC = 0.87 (0.81 – 0.92), accuracy = 78%, sensitivity = 78%, specificity = 75%); however, the ET classifier showed the highest predictive ability (AUC = 0.99 (0.97 – 1), accuracy = 96%, sensitivity = 96%, specificity = 95%), followed by RF classifier (AUC = 0.98 (0.97 – 1.00), accuracy = 92%, sensitivity = 92%, specificity = 92%). Although the predictive performance of ET did not significantly differ from either RF, XGB, or LGB (Delong’s test *P* = 0.10 – 0.16; Fig. 6), it was significantly better compared with the single-model classifiers (Delong’s test *P* < 0.04; Fig. 6).

**Table 2:** Predictive performance of machine learning models for differentiating hemorrhagic ovarian cysts from endometriomas using MRI texture analysis–derived radiomics.

**Performance** **Metrics†**	**ROC-AUC**	**PR-AUC**	**Accuracy**	**F1 Score**	**Precision** **(PPV)**	**Sensitivity** **(Recall)**	**Specificity**	**Brier Score**
**Models**								
**Logistic Regression (LR)**	0.91 (0.86–0.95)	0.90 (0.84–0.95)	81%	81%	82%	81%	79%	0.13
**Naïve Bayes (NB)**	0.87 (0.81–0.92)	0.87 (0.81–0.92)	78%	77%	80%	78%	75%	0.15
**K-Nearest Neighbors (KNN)**	0.96 (0.94–0.98)	0.96 (0.93–0.98)	88%	88%	88%	88%	87%	0.08
**Support Vector Machine (SVM)**	0.95 (0.92–0.97)	0.94 (0.90–0.97)	85%	85%	85%	85%	85%	0.10
**Random Forest (RF)**	0.98 (0.97–1.00)	0.98 (0.96–0.99)	92%	92%	92%	92%	92%	0.06
**Extra Trees (ET)**	0.99 (0.97–1.00)	0.99 (0.97–1.00)	96%	96%	96%	96%	95%	0.05
**XGBoost (XGB)**	0.97 (0.95–0.99)	0.96 (0.92–0.99)	91%	91%	91%	91%	91%	0.07
**LightGBM (LGB)**	0.97 (0.94–0.99)	0.95 (0.89–0.99)	92%	92%	92%	92%	92%	0.07

† ROC-AUC: Area under the receiver operating characteristic curve, PR-AUC: Area under the precision-recall curve, PPV: Positive predictive value. Each of ROC-AUC, PR-AUC, accuracy, F1, precision, sensitivity, and specificity values is expressed as a weighted average.

†† The values within the parentheses represent the 95% confidence intervals (95% CI)

**Fig. 7:**
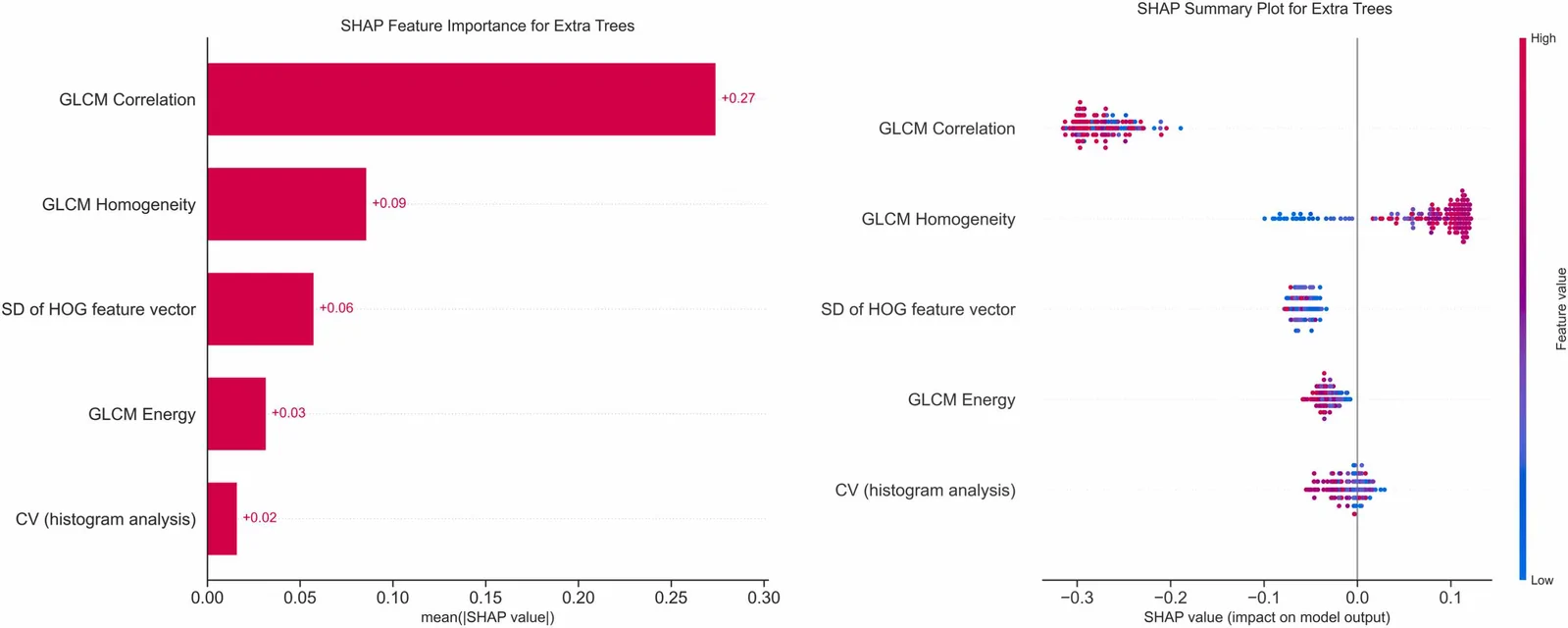
SHapley Additive exPlanations (SHAP) feature importance (***Left***) and SHAP summary (***Right***) plots for the classifier with the highest predictive performance (Extra Trees (ET)).

Fig. 7 depicts the SHAP feature importance and summary plots for the ET classifier. It is evident that GLCM correlation and GLCM homogeneity (similar to other ensemble models, including RF, XGB, and LGB; Supplementary Figure S1) contributed more significantly to the final model output, underscoring the role of texture analysis radiomics in the differentiation of endometrioma and HOC.

## 4 Discussion

This study demonstrated that combining MRI-based radiomics with ML can effectively distinguish endometriomas from HOCs. Among the tested ML models, ensemble models, particularly ET and RF (ROC-AUC scores: 0.99 and 0.98; sensitivities: 96% and 92%; specificities: 95% and 92%, respectively), performed exceptionally well. These results suggested that radiomics-based quantitative imaging markers, especially texture and gradient features, can detect subtle differences in lesion heterogeneity and internal structure that are not easily visible through visual inspection, even by highly experienced radiologists.

Notably, in contrast to ET and other well-performing ML algorithms, the NB classifier showed the lowest predictive performance in this study. Although NB models are computationally efficient and perform well in certain high-dimensional datasets, they assume conditional independence between features [15]. Therefore, NB optimality depends critically on how dependencies among features distribute across classes. Specifically, NB remains optimal when dependencies are evenly distributed between classes or when they cancel each other out [16,17]. Radiomic features, particularly those derived from texture matrices and gradient descriptors, are inherently correlated due to their mathematical derivation from shared pixel intensities [18,19]. Although the selected radiomics features in our analysis were not strongly correlated (Fig. 4), these features might have exhibited dependence structures that were neither perfectly balanced nor self-canceling across the two lesion types. Ensemble methods such as ET and RF, which do not rely on independence assumptions, were consequently better able to capture the complex, interacting patterns in the feature space, leading to their superior performance [20,21].

The findings of this study align with prior research emphasizing the importance of quantitative MRI analysis in characterizing adnexal lesions. Earlier studies have indicated that traditional MRI features, such as T1 hyperintensity and T2 shading, often overlap between endometriomas and HOCs, making differentiation difficult [22]. Quantitative methods, including diffusion-weighted imaging (DWI) and ADC measurements, have shown improved ability to distinguish them, with endometriomas generally displaying lower ADC values than hemorrhagic cysts [23]. More recently, MRI texture analysis research has found that radiomic features can surpass traditional qualitative signs, offering greater diagnostic accuracy and objectivity [24]. Our results build upon this evidence by demonstrating that radiomics features can further improve diagnostic performance.

These findings are especially relevant when considering real-world clinical practice and comparing them to standard imaging interpretation. Conventional MRI for endometriosis typically offers sensitivity and specificity around 86.7% and 81.9%, respectively [25], suggesting that radiomics-based models could outperform traditional qualitative assessments by capturing subtle texture details beyond human perception. Ultrasound radiomics research further indicates that these models can match the diagnostic accuracy of experienced radiologists, significantly supporting clinicians when used as decision aids [[26], [27], [28]]. This shows that radiomics complements expert visual analysis, helping to reduce variability between observers and create more consistent interpretations. While radiomics models often display both high sensitivity and specificity, traditional methods usually favor one depending on clinical priorities. Nevertheless, a key challenge for real-world use involves variability in MRI protocols, sequences, and scanner types, which can affect the stability and reproducibility of radiomic features [2,24]. Addressing this through harmonization is essential before broad clinical implementation.

Beyond MRI, evaluating adnexal masses involves multimodal and hybrid methods. Ultrasound remains the primary choice due to its accessibility and high sensitivity, while MRI offers better tissue characterization. CT has a more limited but supportive role in complex or cancer-related cases. Radiomics can be utilized across all these imaging techniques, with studies showing high performance in ultrasound- and CT-based radiomics for gynecologic tumors, often with AUCs over 0.95. Combining radiomics with clinical data, such as age, symptoms, and CA-125, into nomograms has been shown to enhance diagnostic accuracy and specificity, sometimes nearly perfect [26]. Additionally, non-radiomics AI techniques, such as deep learning (DL) models, have shown strong results in classifying ovarian lesions, sometimes matching or surpassing expert radiologists by capturing complex spatial features without manual feature extraction [29]. Emerging evidence also supports hybrid approaches that integrate radiomics, DL outputs, and structured reporting tools, such as O-RADS, leading to improved sensitivity and specificity [30]. Ultimately, the most impactful clinical approach likely involves an integrated AI ecosystem combining radiomics, DL, and clinical-imaging biomarkers to provide robust, generalizable, and interpretable decision support across various imaging environments.

### 4.1 Limitations and Future Directions

Several limitations should be considered when interpreting these findings. First, although the sample size exceeds that of many previous radiomics studies and the single-scanner, single-vendor design reduces acquisition heterogeneity and strengthens internal consistency, the absence of external, multicenter validation limits the generalizability; differences in MRI acquisition protocols, scanner types, and reconstruction parameters can significantly affect the reproducibility of radiomic features. Therefore, in the absence of external validation, which is a central limitation in this study, definite clinical implications remain to be further investigated. Additionally, differential verification bias cannot be excluded, as most HOCs were confirmed by follow-up MRI, whereas most endometriomas were confirmed by surgery and histopathology. This difference in reference standards may also limit the generalizability of the reported diagnostic performance. Second, our study used a one-slice T2W MRI for radiomics extraction and analysis, consistent with the benefits reported in the scholarly literature. Although previous studies have demonstrated the validity and efficiency of one-slice [31] and a few slice-based [32] segmentations to lower annotation costs while maintaining relatively the same performance for radiomics analysis, three-dimensional acquisition could potentially provide more comprehensive textural data from the entire lesion and ensure higher reproducibility. Moreover, radiomics analysis relies heavily on lesion segmentation, which in this study was manually performed by a single radiologist. No intra-observer or inter-observer reproducibility analysis was carried out; thus, it is susceptible to inter-observer variability. While the use of standardized preprocessing and feature selection techniques helps to reduce some variability, full standardization remains a challenge. Segmentation reliability should also be regarded as an unassessed source of potential bias in the reported feature stability and model performance. Future studies should address these limitations by incorporating prospective, multicenter datasets, formal segmentation reproducibility assessment, examining the performance of these models through external validation, implementing fully automated segmentation pipelines on three-dimensional volumetric acquisitions, and applying harmonization techniques to enhance reproducibility across imaging platforms.

## 5 Conclusion

In summary, MRI-based radiomics combined with ML algorithms provides a highly accurate, non-invasive approach for differentiating endometriomas from HOCs. The superior performance of ensemble-based classifiers, particularly ET and RF, driven by texture features, underscores the potential of radiomics to eventually complement and enhance conventional radiological assessment. From a clinical perspective, and pending external, multicenter, reader-inclusive validation, this technique may improve diagnostic confidence, reduce the number of unnecessary surgical interventions, and support more informed patient management.

## CRediT authorship contribution statement

**Armin Doostparast:** Writing – review & editing, Writing – original draft, Visualization, Validation, Supervision, Software, Resources, Project administration, Methodology, Investigation, Formal analysis, Data curation, Conceptualization. **Zahra Alami:** Writing – review & editing, Writing – original draft, Validation, Supervision, Project administration, Methodology, Investigation, Data curation, Conceptualization. **Sajjad Sadeghpour:** Writing – review & editing, Writing – original draft. **Maryam Ghandhari:** Writing – review & editing, Writing – original draft, Validation, Software, Project administration, Methodology, Data curation. **Mohamad Amin Bakhshali:** Writing – review & editing, Supervision, Formal analysis. **Parvaneh Layegh:** Writing – review & editing, Supervision, Resources, Project administration, Methodology, Investigation, Conceptualization.

## Ethics declaration

Written informed consent to take part in the study and to publish the article has been obtained from all participants or their legal representatives. The privacy rights of participants have been observed.

This study included organ or tissue donors. This study includes human biological material and consent was obtained by donors, or their next of kin or legal representatives, for use in this study and for publication of the article. The samples used in this research were not sourced from executed prisoners or prisoners of conscience.

This study was performed in compliance with relevant laws, regulatory frameworks and guidelines where the research took place. This study was approved by the Institutional Review Board of Mashhad University of Medical Sciences. (Approval No. IR.MUMS.MEDICAL.REC.1401.566)

## Ethics and consent to participate

The Institutional Review Board of Mashhad University of Medical Sciences approved this study (approval code: IR.MUMS.MEDICAL.REC.1401.566). Written informed consent was obtained from all participants or their legal guardians. All procedures were performed in accordance with the ethical principles of the Declaration of Helsinki.

## Disclosure of financial and proprietary interests for all authors

In accordance with ethical standards and transparency practices, all authors involved in this study have disclosed their financial and proprietary interests. Each author has provided a detailed account of any potential conflicts of interest or explicitly stated that they have no such interests to declare.

## Funding

No funding was received for this study.

## Declaration of Competing Interest

The authors declare that they have no known competing financial interests or personal relationships that could have appeared to influence the work reported in this paper.

## Data Availability

The datasets generated and/or analyzed during the current study are not publicly available due to institutional data-sharing policy restrictions on patient-derived data, but could be obtained from the corresponding author upon reasonable request.

## Appendix A Supplementary material

Supplementary material

supplementary Figure S1 SHapley Additive exPlanations (SHAP) feature importance (***Left***) and SHAP summary (***Right***) plots for all eight classifiers (LR, NB, KNN, SVM, RF, ET, XGB, LGB)
