# Supplementary material for: MRI-based radiomics analysis and machine learning in the differentiation of endometriomas from hemorrhagic ovarian cysts
Source: Eur J Radiol Open. 2026 Sep 12;17:100818. doi: 10.1016/j.ejro.2026.100818 (PMC13586818; doi:10.1016/j.ejro.2026.100818)
Supplement: Supplementary file 1 [file mmc1.docx]

| **Supplementary Table S1.** Summary of extracted radiomic features | |
| --- | --- |
| Feature Category | Feature List |
| First-order statistics (n = 20) | Energy, Total Energy, Entropy, Minimum, 10th Percentile, 90th Percentile, Maximum, Mean, Median, Interquartile Range (IQR), Range, Mean Absolute Deviation (MAD), Robust Mean Absolute Deviation (rMAD), Root Mean Squared (RMS), Standard Deviation (SD), Skewness, Kurtosis, Variance, Uniformity, Coefficient of Variation (CV) |
| Shape features (n = 10) | Mesh Surface, Pixel Surface, Perimeter, Perimeter to Surface Ratio, Sphericity, Spherical Disproportion, Maximum 2D Diameter, Major Axis Length, Minor Axis Length, Elongation |
| Gray Level Co-occurrence Matrix (GLCM) (n = 24) | Autocorrelation, Joint Average, Cluster Prominence, Cluster Shade, Cluster Tendency, Contrast, Correlation, Difference Average, Difference Entropy, Difference Variance, Joint Energy, Joint Entropy, Informational Measure of Correlation (IMC1), Informational Measure of Correlation (IMC2), Inverse Difference Moment (IDM), Maximal Correlation Coefficient (MCC), Inverse Difference Moment Normalized (IDMN), Inverse Difference (ID), Inverse Difference Normalized (IDN), Inverse Variance, Maximum Probability, Sum Average, Sum Entropy, Sum of Squares (Variance) |
| Gray Level Size Zone Matrix (GLSZM) (n = 16) | Small Area Emphasis (SAE), Large Area Emphasis (LAE), Gray Level Non-Uniformity (GLN), Gray Level Non-Uniformity Normalized (GLNN), Size-Zone Non-Uniformity (SZN), Size-Zone Non-Uniformity Normalized (SZNN), Zone Percentage (ZP), Gray Level Variance (GLV), Zone Variance (ZV), Zone Entropy (ZE), Low Gray Level Zone Emphasis (LGLZE), High Gray Level Zone Emphasis (HGLZE), Small Area Low Gray Level Emphasis (SALGLE), Small Area High Gray Level Emphasis (SAHGLE), Large Area Low Gray Level Emphasis (LALGLE), Large Area High Gray Level Emphasis (LAHGLE) |
| Gray Level Run Length Matrix (GLRLM) (n = 16) | Short Run Emphasis (SRE), Long Run Emphasis (LRE), Gray Level Non-Uniformity (GLN), Gray Level Non-Uniformity Normalized (GLNN), Run Length Non-Uniformity (RLN), Run Length Non-Uniformity Normalized (RLNN), Run Percentage (RP), Gray Level Variance (GLV), Run Variance (RV), Run Entropy (RE), Low Gray Level Run Emphasis (LGLRE), High Gray Level Run Emphasis (HGLRE), Short Run Low Gray Level Emphasis (SRLGLE), Short Run High Gray Level Emphasis (SRHGLE), Long Run Low Gray Level Emphasis (LRLGLE), Long Run High Gray Level Emphasis (LRHGLE) |
| Neighboring Gray Tone Difference Matrix (NGTDM) (n = 5) | Coarseness, Contrast, Busyness, Complexity, Strength |
| Gray Level Dependence Matrix (GLDM) (n = 14) | Small Dependence Emphasis (SDE), Large Dependence Emphasis (LDE), Gray Level Non-Uniformity (GLN), Dependence Non-Uniformity (DN), Dependence Non-Uniformity Normalized (DNN), Gray Level Variance (GLV), Dependence Variance (DV), Dependence Entropy (DE), Low Gray Level Emphasis (LGLE), High Gray Level Emphasis (HGLE), Small Dependence Low Gray Level Emphasis (SDLGLE), Small Dependence High Gray Level Emphasis (SDHGLE), Large Dependence Low Gray Level Emphasis (LDLGLE), Large Dependence High Gray Level Emphasis (LDHGLE) |
| Histogram of Oriented Gradients (HOG) (n = 2) | HOG_mean, HOG_stdⁱ |
| Total (n = 107) |  |
| ⁱ HOG features were extracted in MATLAB R2020b using the following parameters: cell size = 8×8 pixels, block size = 2×2 cells, block stride = 8 pixels, and number of orientation bins = 9. This configuration yielded a 3,780-dimensional feature vector per image. For subsequent statistical analysis, the mean and standard deviation of this high-dimensional HOG descriptor were computed, resulting in two aggregate features (HOG_mean and HOG_std), representing the central tendency and variability of the local gradient distributions.  ⁱⁱ Except for HOG, the other radiomics features were computed in Pyradiomics 3.0.1. Gray-level discretization for the texture matrices was performed using a fixed bin width of 25.  *Abbreviations: GLCM = Gray Level Co-occurrence Matrix, GLRLM = Gray Level Run Length Matrix, GLSZM = Gray Level Size Zone Matrix, NGTDM = Neighboring Gray Tone Difference Matrix, GLDM = Gray Level Dependence Matrix, HOG = Histogram of Oriented Gradients, n = number, 2D = two-dimensional, IQR = Interquartile Range, MAD = Mean Absolute Deviation, rMAD = Robust Mean Absolute Deviation, RMS = Root Mean Squared, SD = Standard Deviation, CV = Coefficient of Variation* | |
